# Dynamic metrics-based biomarkers to predict responders to anti-PD-1 immunotherapy

**DOI:** 10.1038/s41416-018-0363-8

**Published:** 2018-12-27

**Authors:** Can Liu, Hua He, Xiaobing Li, Maureen A. Su, Yanguang Cao

**Affiliations:** 1 Division of Pharmacotherapy and Experimental Therapeutics, Eshelman School of Pharmacy, University of North Carolina at Chapel Hill, Chapel Hill, NC 27599 USA; 2 School of Pharmacy, China Pharmaceutical University, 210009 Nanjing, Jiangsu China; 3 Department of Pharmacy, Shengjing Hospital of China Medical University, 110004 Shenyang, Liaoning China; 4 Department of Microbiology, Immunology and Medical Genetics (MIMG) and Pediatrics, University of California, Los Angeles, CA 90095 USA; 5 Lineberger Comprehensive Cancer Center, University of North Carolina at Chapel Hill, Chapel Hill, NC 27599 USA

**Keywords:** Cancer immunotherapy, Cancer immunotherapy, Statistical methods, Cancer immunotherapy, Statistical methods

## Abstract

**Background:**

Anti-PD-1 immunotherapies have shown clinical benefit in multiple cancers, but response was only observed in a subset of patients. Predicting which patients will respond is an urgent clinical need, but current companion diagnosis based on PD-L1 IHC staining shows limited predictability.

**Methods:**

A dynamic, metrics-based biomarker was developed to discriminate responders from non-responders for anti-PD-1 immunotherapy in B16F10 melanoma-bearing mice.

**Results:**

Similar to patients, there was considerable heterogeneity in response to anti-PD-1 immunotherapy in mice. Compared with the control group, 45% of anti-PD-1 antibody-treated mice displayed improved survival (defined as responders) and the remainder only gained little, if any, survival benefit from PD-1 blockade (non-responders). Interestingly, the dynamics of IFN-γ secretion by peripheral lymphocytes was associated with faster secretion onset (shorter lag time), stronger exponential phase, shorter time to half magnitude, and higher magnitude of secretion in responders at day 10 after tumour inoculation. To sufficiently predict responders from non-responders, IFN-γ secretion descriptors as well as phenotypic markers were subjected to multivariate analysis using orthogonal partial least-squares discriminant analysis (OPLS-DA).

**Conclusions:**

By integrating phenotypic markers, IFN-γ secretion descriptors sufficiently predict response to anti-PD-1 immunotherapy. Such a dynamic, metrics-based biomarker holds high diagnostic potential for anti-PD-1 checkpoint immunotherapy.

## Introduction

Immune checkpoint proteins (such as PD-1/PD-L1, CTLA-4/B7-1) are critical molecules governing tumour immune-surveillance and antitumour response. Tumour cells can escape immune eradication by upregulating inhibitory checkpoint molecules (such as PD-L1), thereby inhibiting cytotoxic T cells, and dampening immune response.^1–3^ Accordingly, tumour immunotherapies that block inhibitory checkpoint pathways have shown prominent clinical benefits with remarkable remission and durable response.^4^ Since the approval of anti-CTLA-4 antibody (ipilimumab) and anti-PD-1 antibodies (pembrolizumab and nivolumab) in the treatment of advanced melanoma, checkpoint inhibitors have shown efficacy in non-small-cell lung cancer, renal cell carcinoma, and Hodgkin lymphoma, and several others.^5^

Multiple clinical trials showed that prolonged response to checkpoint immunotherapy was only observed in 20–40% of patients, and majority of patients were either poor or none responders.^6–8^ So far, PD-L1 IHC staining is the only “complementary diagnostic” approved by US FDA to pre-screen potential responders. However, PD-L1 IHC staining is extremely variable with high rates of false positive, which limits its diagnostic utility.^9^ Variable expression of PD-L1 was observed from different anatomical sites in individual patients and even for the same anatomical site there was high variability in multiple tumour biopsies collected over time.^8,9^ A meta-analysis including 20 trials showed that a significant fraction of PD-L1-negative patients still responded to anti-PD-1 treatments.^10^ Non-responders are needlessly subjected to the high cost and excessive toxicity of checkpoint inhibitors, and thereby sufficient diagnostic biomarkers are urgently needed to guide patient selection.

Considering that tumour PD-L1 expression is heterogeneous, inducible, and evolving,^3,8,9^ it is almost impossible to accurately predict the dynamic immune reactivity by “time-frozen snapshots” of biomarkers, like PD-L1 IHC staining. So far, almost all putative biomarkers, such as mutational landscape and intra-tumour lymphoid infiltrates, are static and none of them has been advanced for diagnostic use yet.^11^ Beyond traditional static biomarkers, dynamic biomarkers, which integrate multifactorial variables and temporal signatures, show high diagnostic potential. A classic dynamic diagnostic that is well-accepted for type II diabetes, for instance, is the oral glucose tolerance test (OGTT). Compared with static fasting glucose, the temporal profiles of plasma glucose in OGTT and the parameters extracted by modelling glucose profiles robustly predict the degree of insulin resistance, and importantly this method is less compromised by sampling time and schedule. Dynamic biomarkers have also been broadly evaluated to monitor disease stages or drug responses in Alzheimer’s disease, infection diseases, and cancers.^12–15^ Recent studies also provided a strong rationale to develop dynamic and network biomarkers to reflect evolving immune functions.^11,16^ For instance, model-based dynamic biomarkers were developed to predict cytotoxic T cell age^17^ and IL-2 production.^18^ Network analysis of gene expression profiling from responders has also been applied to optimise therapeutic combinations with CTLA-4 blockade.^19^

Here we develop a new type of dynamic biomarker for anti-PD-1 immunotherapy, which is based on the interferon gamma (IFN-γ) cytokine secretion kinetics of circulating lymphocytes in the peripheral blood, to reflect evolving immune functions and further predict individual responsiveness to checkpoint immunotherapies.^20,21^ Specifically, the secretory kinetics of IFN-γ, rather than concentration measurements at a single time point, are evaluated in peripheral CD4+ and CD8+ T cells from melanoma-bearing mice during the treatment of anti-PD-1 immunotherapy. This strategy shows potential to capture small, latent but intrinsically evolving nature of T cells in response to immune perturbations.^22^ Secretion descriptors (such as magnitude, slope, lag time, and time to half magnitude), after integrating phenotypic markers, were further analysed using orthogonal partial least-squares discriminant analysis (OPLS-DA) to adequately discriminate responders from non-responders to anti-PD-1 immunotherapy.

## Materials and methods

### Mice

Wild-type C57BL/6 mice (7- to 9-week-old-female) were purchased from Jackson Laboratories (Bar Harbor, ME). Animal care was conducted in accordance with institutional guidelines at School of Pharmacy, UNC-Chapel Hill. Experimental procedures were approved by Institutional Animal Care and Use Committee of UNC-Chapel Hill.

### Antibodies

Control rat IgG (2A3) and anti-PD-1 (RMP1-14) used in vivo were obtained from BioXcell. Dosing per injection was 250 μg of 2A3 and 250 μg of RMP1-14.

Staining antibody included FITC anti-CD45.2 (104), PE-Cy7 anti-CD4 (RM4-5), PerCP-Cy5.5 anti-CD8a (53-6.7), BV421 anti-PD-1 (J43) from BD Biosciences; APC anti-CD25 (PC61.5), PE anti-Foxp3 (FJK-16s), and PE-Cy5 anti-CD11b (M1/70) from eBiosciences, and APC-Cy7 anti-Gr-1 (RB6-8C5) from Thermo Scientific.

### Tumour model and treatment regimens

**Fig. 1:**
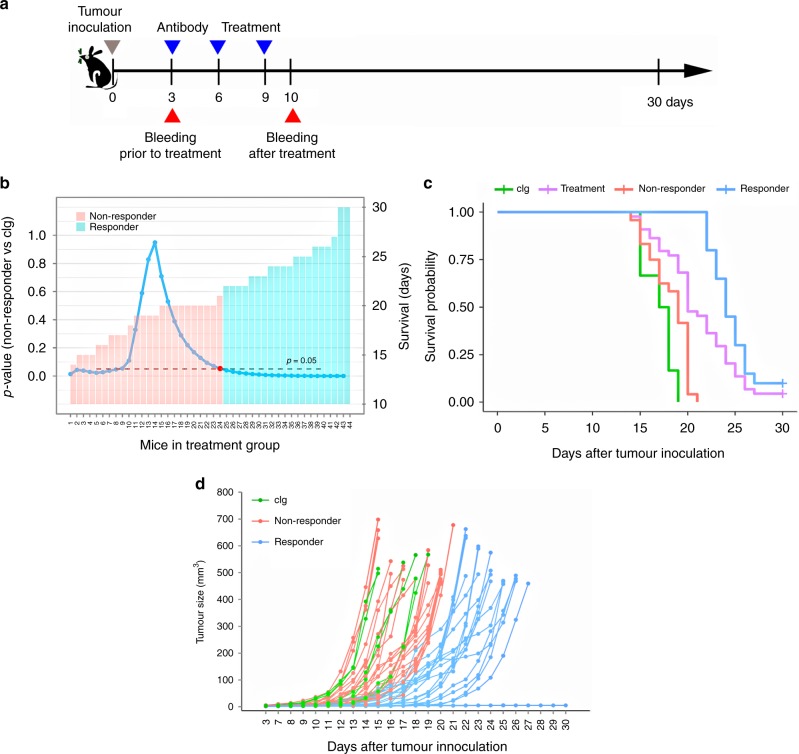
**a** Schematic diagram of immunotherapy regimen and sampling strategy on mice challenged with 5 × 10^4^ B16F10 cells. **b** Scanning cut-off for stratification of responders and non-responders to anti-PD-1 treatment. Histogram indicates individual survival time following anti-PD-1 treatment. Survival differences between non-responder and untreated groups were analysed by Log-rank test. *P*-value at each step was showed as line chart. Red dot indicates the best cut-off. **c** Kaplan–Meier survival curves of mice according to different treatment (cIg vs treatment). Responder and non-responder are sub-groups of PD-1 treatment. Log-rank test was used to compare survival curves. **d** Tumour growth following B16F10 inoculation. Tumour sizes are individually represented over time

B16F10 mouse melanoma cell line of C57BL/6 origin was purchased from ATCC. As shown in Fig. 1a, mice underwent B16F10 injection (5 × 10^4^ cells, s.c.) in the flank at day 0. Visible tumours were confirmed at day 3 in mice prior to randomisation into different groups. Mice were treated at days 3, 6, and 9, respectively, with indicated therapeutic antibody i.p.^23^ Peripheral blood (110 μl) was collected at days 3 (prior to treatment) and 10 (one day after final treatment). Tumours were daily measured by a calliper. For cell phenotyping within tumours in an independent experiment, mice were sacrificed, and tumour samples were harvested at day 17.

### Cell phenotyping

Cell frequencies including CD4+ lymphocytes, CD8+ lymphocytes, CD25+Foxp3+ regulatory T cells (Treg), PD-1+ lymphocytes in peripheral and tumour-infiltrating lymphocytes (TILs) were determined by flow cytometry. Gr-1+CD11b+ myeloid-derived suppressor cells (MDSC) were also determined peripherally. Whole blood (10 μl) was directly processed for staining following the lysis of erythrocytes (ACK lysis buffer; Gibco). In the case of tumour,^24^ fresh excised tumours were digested by Collagenase/Dispase and DNase (Roche). After removal of erythrocytes, TILs were enriched by a ficoll gradient (Histopaque-1077; Sigma). For surface staining, cell suspensions were stained with indicated staining antibodies in the presence of anti-CD16/32 (BD Biosciences). For intracellular staining, cells were fixed, permeabilised using Foxp3/transcription factor staining buffer set (eBiosciences) prior to staining. LIVE/DEAD fixable yellow dead cell stain kit (Thermofisher scientific) was used to exclude dead cells. Stained cells were acquired on an LSRII cytometer (BD Biosciences).

### IFN-γ secretion dynamics by peripheral lymphocytes

Peripheral blood mononuclear cells (PBMCs) were isolated from 100 μl peripheral blood using ficoll gradient. CD4+ and CD8+ lymphocytes were simultaneously sorted from PBMC by FACS. Purified lymphocytes were stimulated by PMA and ionomycin (eBioscience) in a 200 μl of RPMI 1640 in a 96-well plate. Cells were then incubated and serial supernatant (100 μl) were collected at 1, 2, 3, 4, 6, 8, 12, 24, and 40 h following stimulation. An equal volume of RPMI 1640 containing stimulus was complemented after each sampling. Serial IFN-γ secretions from peripheral CD4+ and CD8+ lymphocytes were quantified by Mouse IFN-γ ELISA Kit II (BD Biosciences).

### Data processing

Dynamic IFN-γ secretion profiles would be described with a Sigmoid *E*_max_model (Hill equation):

1 $$Y\left( t \right) = \frac{{C_{{\mathrm {max}}} \cdot t^h}}{{{\mathrm {Tc50}}^h + t^h}},$$

where *Y*(*t*) means the predicted concentration at time *t*, *C*_max_ is the maximum IFN-γ concentration, Tc50 represents the time to reach 50% *C*_max_, and *h* is a slope factor (Hill coefficient). In addition, tau is the lag time of IFN-γ secretion: only if *t* > tau, *Y*(*t*) fits Eq. (1), otherwise *Y*(*t*) = 0.

Secretion descriptors such as *C*_max_, Tc50, *h*, and tau were estimated by ADAPT 5 with the STS method (http://bmsr.usc.edu/Software/ADAPT/). The IFN-γ data were fitted simultaneously using maximum likelihood estimation with following variance model:

2 $${\mathrm {Var}}\left( t \right) = \left( {\sigma _1 + \sigma _2 \times Y\left( t \right)} \right)^2,$$

where Var(t) is the variance of the concentration at a specific time point, and *σ*_1_ and *σ*_2_ are the additive and proportional variance parameters.

### Multivariate analysis

Orthogonal partial least-squares discriminant analysis (OPLS-DA) was applied to project multi-variables to distinguish responders and non-responders. OPLS-DA is a supervised multivariate regression method to return a dimension reduction to relate a categorical response matrix *Y* (in our case, responder and non-responder) to a predictor matrix *X* (e.g. *C*_max_, Tc50, *h*, tau, and other variables). OPLS-DA is an extension to the PLS-DA regression method featuring an integrated orthogonal signal correction filter. As a result, systematic variation in *X* is separated into predictive (correlated to *Y*) and orthogonal (uncorrelated to *Y*) information. This leads to an equal predictive capacity compared to PLS-DA but dramatically facilitates the model interpretation.^25,26^

The predictor matrix *X* includes IFN-γ secretion descriptors derived from CD4+ (CD4Cmax, CD4Tc50, CD4h, CD4tau) and CD8+ (CD8Cmax, CD8Tc50, CD8h, CD8tau) T cells or/and phenotypic markers (CD4+, CD8+, PD1+CD4+, PD1+CD8+, Treg, CD8+/Treg, MDSC) from peripheral lymphocytes phenotyping. Data standardisation (mean-centred and unit-variance scaled) was performed to all variables and subjected to model building. The unsupervised segregation was checked by principal components analysis (PCA) prior to OPLS-DA.^27^

*R*^2^*X* and *R*^2^*Y* are equivalent to the fraction of *x* and *y*-variation modelled in the component, respectively, which are related to the model’s goodness of fit. To assess the model predictive performance, seven-fold cross-validation was conducted and *Q*^2^*Y* represented overall cross-validated *R*^2^*Y* for the component. Model performance was further validated by randomly permuting the samples 200 times and recalculating derived *R*^2^ and *Q*^2^.^28^ Models were considered acceptable if *R*^2^ and *Q*^2^ significantly degraded with sample permutation. The dataset used for external validation was from an independent preliminary experiment performed prior to the current study. The dataset contained 20 melanoma-bearing mice with anti-PD-1 treatment, where MDSC% was not considered as a phenotypic parameter. A similar cut-off scanning method as Fig. 1b was used to stratify responders and non-responders contingent on the tumour growth.

Variable influence on projections (VIP), a parameter that reveals the importance of the *X* variables for both the *Y* matrix and model components, is used for variable selection. Preliminary OPLS-DA models were carried out first with all variables and VIP was assessed for each variable. We then performed variable selection prior to final analysis by removing variables with VIP < 0.4. The number of predictive and orthogonal components were both set to 1 according to *Q*^2^*Y* assessment. Statistical analysis and plotting were done by R (version 3.3.2) with *ropls* package.

## Results

### Checkpoint blockade improved survival with considerable heterogeneity

B16F10 induced melanoma is partial immunogenic to immunotherapies. A relatively low number of cells (5 × 10^4^ cells) was inoculated into C57BL/6 mice to induce melanoma. Tumour inoculation and antibody treatment schema are summarised in Fig. 1a. Lack of survival was defined as death or tumour size reaching 500 mm^3^. Observation ended at day 30 when almost all mice reached the endpoint. All control mice (cIg group) died before day 19 following inoculation (Fig. 1c). While there was a modest increase in survival, there was also considerable heterogeneity in response to anti-PD-1 antibody treatment.

We stratified mouse populations into responders and non-responders based on their survival status. Mice from the PD-1-treated group were pooled and sorted by survival duration (Fig. 1b). To scan for the best separation cut-off, we initially put mice with the shortest survival into the non-responder group and others into the responder group. We then added mice into the non-responder group one by one to calculate *P*-values between the non-responder and the control group at each step by log-rank test. The lowest *P*-value above 0.05 indicating no better survival than the control group was then defined as the best cut-off, which was marked as red dot (Fig. 1b). As indicated, 24 out of 44 mice are non-responders with no longer than 21 days’ survival. Figure 1c shows overall survival of responders and non-responders. After stratification, the PD-1 treatment group was divided into two sub-groups: non-responders showed similar survivals with the control group while responders gained significant therapeutic benefit. In the context of individual profiles (Fig. 1d), apparent tumour progression could be observed in non-responders since day 12. Before that, responders and non-responders exhibited indistinguishable tumour growth curves.

### Phenotypic parameters are not satisfactory biomarkers

In the tumour microenvironment, modulation of T cell infiltration by checkpoint blockade was evaluated in an independent experiment (Fig. S1). Responders were associated with higher CD8+ cells but lower CD4+ cells infiltration, lower Foxp3 + Treg density and higher CD8+ to Treg ratio (Fig. S1, responder vs non-responder), indicating strongly enhanced T effector function within the tumours.

**Fig. 2:**
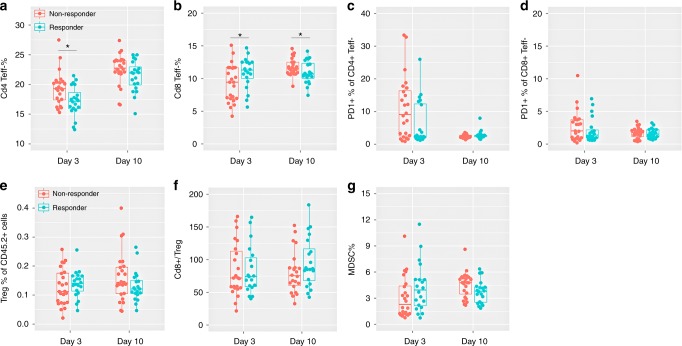
Cell phenotyping on peripheral lymphocytes at day 3 and day 10. **a**, **b** Per cent of CD4+ (**a**) and CD8+ (**b**) T effector cells of CD45.2+ cells. **c**, **d** Frequency of PD-1+ subset in CD4+ (**c**) and CD8+ (**d**) T cells. e Per cent of CD25+Foxp3+ regulatory T cells (Treg) of CD45.2+ cells. **f** CD8+ T cells to Treg ratio. **g** Frequency of MDSCs. Values shown are for individually analysed mice. Data are analysed by Wilcoxon–Mann–Whitney test (*p < 0.05)

However, these phenotypic features are characterised at late stage (day 17) and are therefore of a minimal predictive value. To identify earlier prognostic biomarkers, circulating lymphocyte phenotyping was carried out at day 3 and day 10 (Fig. 2). Representative dot plots are shown in Supplementary Fig. S2. Compared with non-responders, responders show a trend to employ lower peripheral CD4+, CD8+, CD25+Foxp3+Treg and Gr-1+CD11b+MDSC cell density, higher CD8+ to Treg ratio, and similar PD-1+ fraction at day 10 (Fig. 2), which is consistent with the trend within tumour tissue except CD8+ cells (Fig. S1). However, responders were characterised as higher CD8+, Treg, and MDSC cells, similar CD8+ to Treg ratio, but lower PD1+ subset in CD4+ cells than non-responders at day 3 (Fig. 2b, c, e–g).

Univariate analysis was assessed on each phenotypic marker. Apart from CD4+% at day 3, CD4+% and CD8+% at day 10, no individual marker was able to differentiate responders and non-responders with statistical significance (*p* < 0.05). Collectively, though peripheral phenotyping may provide some insights into immune response, they are not sufficient to be independently used as predictive biomarkers.

### Diagnostic potential of IFN-γ secretion by peripheral lymphocytes

**Fig. 3:**
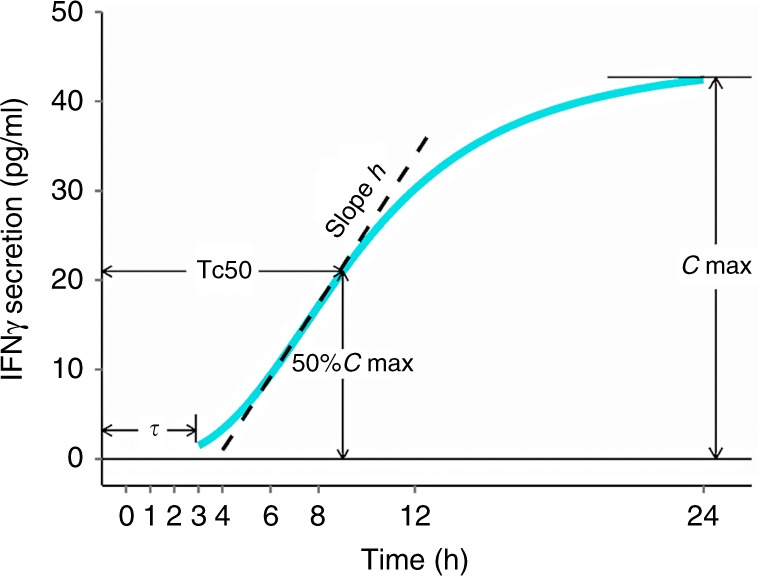
Graphical representation of IFN-γ secretion descriptors derived from simulated curves. *C*_max_, Tc50, Hill exponent (*h*), and *τ* (tau) indicate magnitude, time to reach 50% *C*_max_, slope, and lag time, respectively

Degrees of cytokine production by lymphocytes are closely associated with disease stage and immune response to infection, cancer, or other immune disorders.^22,29,30^ The cytokine secretion kinetics (rate and duration) also largely reflects the functionality and adaptability of the immune system. We therefore measured IFN-γ secretion kinetics by peripheral T cells over time and extracted multiple descriptors from the secretion profiles. As shown in Fig. 3, magnitude (*C*_max_), time to reach 50% magnitude (Tc50), slope (hill exponent, *h*), and lag time (tau) were extracted from secretion curves to describe not only the amplitude of IFN-γ production but also when and how rapidly T cells respond to stimulus.

**Fig. 4:**
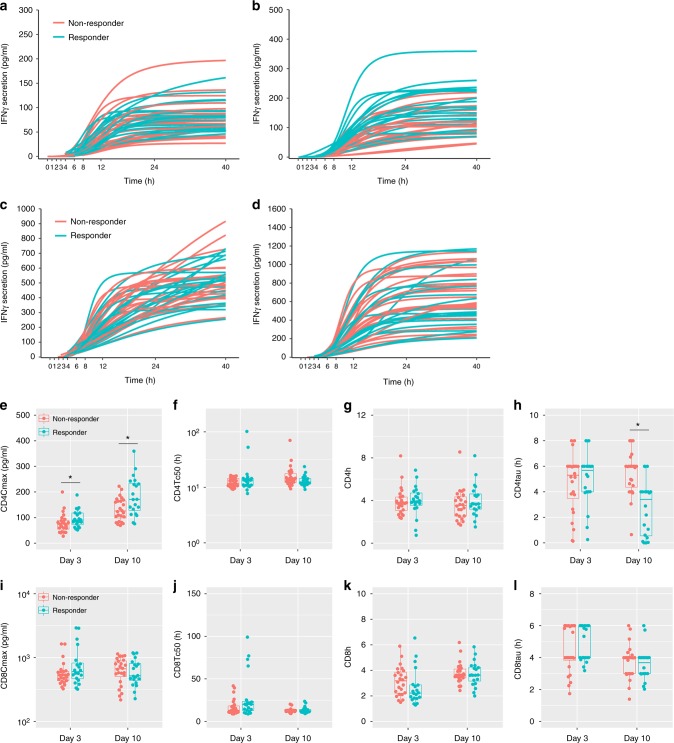
Dynamic IFN-γ secretion from peripheral CD4+ and CD8+ lymphocytes. **a**–**d** Simulated IFN-γ secretion based on Sigmoid *E*_max_ model from CD4+ (**a**, **b**) and CD8+ (**c**, **d**) T cells at day 3 (**a**, **c**) and day 10 (**b**, **d**). Each line represents individual mouse. Dynamic IFN-γ secretion was determined at day 3 and day 10. Supernatant IFN-γ was quantified by ELISA at 1, 2, 3, 4, 6, 8, 12, 24, and 40 h following T cell activation. **e**–**l** Model-based secretion descriptors from CD4+ (**e**–**h**) and CD8+ (**i**–**l**) T cells addressing magnitude (*C*_max_) (**e**, **i**), time to reach 50% *C*_max_ (Tc50) (**f**, **j**), slope (*h*) (**g**, **k**), and lag time (tau) (**h**, **l**) of dynamic IFN-γ secretion

More specifically, IFN-γ secretion dynamics was determined from activated peripheral CD4+ and CD8+ lymphocytes at multiple time points at day 3 and day 10. As shown in Fig. 4a–d, secretory profiles were adequately captured using the sigmoid *E*_max_ model with acceptable goodness of fittings (Fig. S3). As expected, CD8+ lymphocytes were more capable of IFN-γ secretion than CD4+ lymphocytes. In addition, IFN-γ secretion rose notably following anti-PD-1 therapy at day 10 compared to those prior to treatment at day 3.

IFN-γ secretion curves for responders and non-responders were overlapping and interweaved. The secretion profiles were then analysed to extract multiple descriptors. The secretion descriptors revealed that on average responders were associated with faster secretion onset (shorter lag time, CD4tau), stronger exponential phase (CD4h), shorter time to half magnitude (CD4Tc50), and higher magnitude (CD4Cmax) of IFN-γ secretion at day 10 (Fig. 4e–h). However, responders merely showed higher magnitude and slightly higher slope at Day 3. Descriptors derived from CD8+ lymphocytes did not show significant difference (Fig. 4i–l). In accordance with phenotypic markers, an individual IFN-γ secretion descriptor cannot be used as a robust biomarker to precisely point out responders from whole population.

### Development of OPLS-DA models to predict responders

**Fig. 5:**
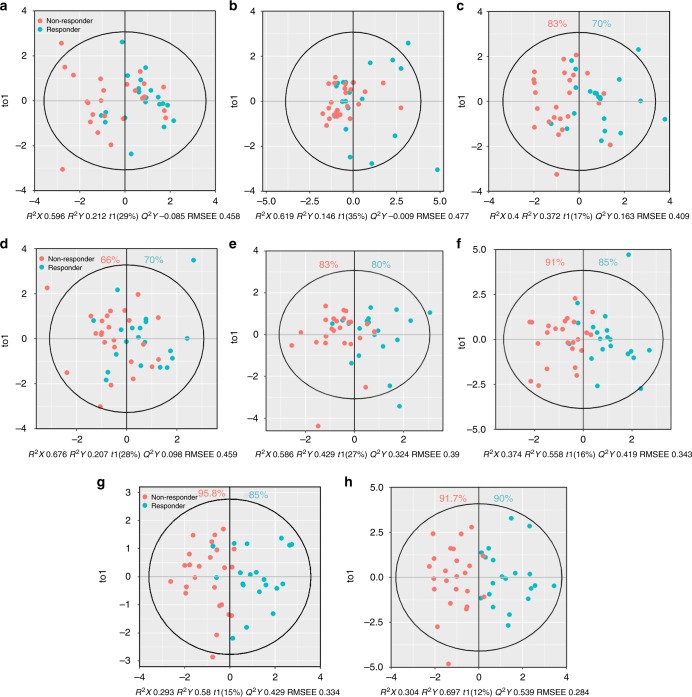
OPLS-DA model outcomes for distinguishing between immune responses. OPLS-DA models were established based on only phenotypic markers (**a**, **d**), only secretion descriptors (**b**, **e**), and all variables (**c**, **f**) at day 3 (**a**–**c**) and day 10 (**d**–**f**). The variable changes from day 3 to day 10 were also used as predicting variables (**g**). All variables at day 3 and day 10 were finally integrated (**h**). The model was generated using one predictive component (*t*1) and the first orthogonal component (*t*o1). Each dot stands for OPLS-DA score of individual mouse. Black hoteling ellipses defined global confidence limits. The values of *R*^2^*X*, *R*^2^*Y*, *Q*^2^*Y,* and RMSEE were shown under each plot. *R*^2^*X* and *R*^2^*Y*: percentage of predictor and response variance explained by the model. *Q*^2^*Y*: predictive performance of the model estimated by cross-validation. RMSEE: root mean square error of estimation. Per cent in colour (only shown when *Q*^2^*Y* is positive) indicates the accurate prediction of responder and non-responder by the model

None of individual variable is sufficient enough for diagnosis, even though some variables predicted significant difference between responders and non-responders. Multivariate models were therefore developed by OPLS-DA. Unlike unsupervised PCA, supervised OPLS-DA is able to maximise covariance between the predictor matrix and the response matrix. We constructed OPLS-DA models based on predictor matrix consisting of phenotypic markers only (Fig. 5a, d), secretion descriptors only (Fig. 5b, e), and all variables (Fig. 5c, f) from day 3 (Fig. 5a–c) and day 10 (Fig. 5d–f).

At day 3, OPLS-DA model generated from phenotypic markers failed to spatially discriminate responders from non-responders (Fig. 5a). Despite the modest separation obtained in the score plot, the *Q*^2^ estimation of predictive performance is extremely low (negative). Interestingly, secretion descriptors-based model showed slightly separation between immune responses with improved but still negative *Q*^2^ (Fig. 5b). With the integration of secretion descriptors and phenotypic markers (Fig. 5c), OPLS-DA model yielded acceptable separation.

At day 10, model derived from phenotypic markers did not achieve good classification (Fig. 5d), but positive *Q*^2^ value indicated a degree of predictability. When IFN-γ secretion descriptors applied (Fig. 5e), the majority of the responders and non-responders clustered in their respective regions with more than 80% accuracy and insignificant overlap. Once phenotypic markers were further included in the model, responders and non-responders were well-clustered with high goodness of fit (*R*^2^) and predictive ability (*Q*^2^) (Fig. 5f). It is demonstrated that multivariate analysis could effectively uncover the underlying predictive potential of the dynamic IFN-γ secretion descriptors by peripheral lymphocytes.

To gain more insights on the immune response to checkpoint inhibitors, the variable changes from day 3 to day 10 were defined as new predictors. Strikingly, these derivative variables also displayed high predictability to individual responses (Fig. 5g). Once all variables at day 3 and day 10 were integrated, we concluded an almost complete distinction with significantly improved data interpretation (*R*^2^*Y* = 0.697) and model predictability (*Q*^2^*Y* = 0.539) (Fig. 5h). VIP plots (Fig. S4B and E) and loading plots (Fig. S4C and F) for both models revealed the significantly contributing variables.

**Fig. 6:**
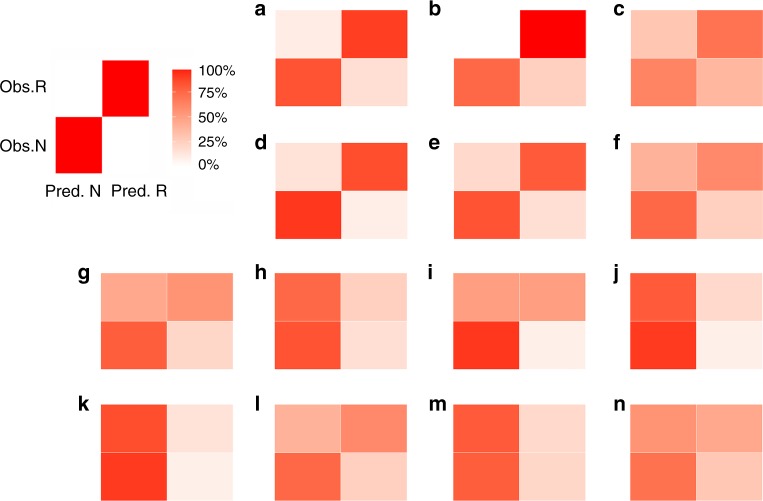
Prediction performance of OPLS-DA models at day 10. Internal validation was conducted on training (**a**) and test (**b**) subset by re-trained all-variable based model at day 10 using training subset. External validation (**c**) was conducted on an independent dataset. Prediction performance of all-variable (**d**), secretion descriptors (**e**) and phenotypic markers (**f)** based multivariate analysis at day 10 were compared. Prediction performance derived from representative univariate analysis (**g**–**n**) were also shown to compare with multivariate analysis. **g**–**n** described univariate analysis of CD4Cmax, CD4h, CD4tau (secretion descriptors of Cmax, h and tau derived from CD4+ lymphocytes), CD8Tc50, CD8tau (secretion descriptors of Tc50 and tau derived from CD8+ lymphocytes), CD8+, PD1+CD4+, MDSC (cell density of CD8+, PD1+% of CD4+ cells, cell density of MDSC in peripheral blood) respectively. Obs. R and Obs. N represent observed responders and non-responders respectively. Pred. R and Pred. N represent predicted responders and non-responders respectively. The diagonal in each panel represents conditions under which the prediction was matched with the observation. Colors indicate the percent (%) of samples that met a given condition. Left-top panel showing an example of prediction with 100% accuracy

To exclude significant separation that was due to data over-fitting, we randomly permuted the response values in the developed models shown in Fig. 5g, h. This reshuffling resulted in coherent decreases in both *R*^2^*Y* and *Q*^2^*Y* (*p* *=* 0.005; Fig. S4A and D), indicating that the developed models were statistically sound. Similar permutations were applied to validate the models shown in Fig. 5c, f (data not shown). In addition to *Q*^2^*Y* value generated by cross-validation and response permutation, the predictive performance of all-variable model on day 10 (Fig. 5f) was further validated by internal and external dataset. Samples on day 10 were randomly and equally separated into training and test subsets. As shown in Fig 6a, b, the model based on the training subset sufficiently predicted the test subset. The predictive accuracy was similar as the model developed with full day-10 dataset (Fig. 6d). External validation of the model was performed with the data from an independent experiment. As shown in Fig. 6c, even in the absence of MDSC values in the external dataset, the model maintained acceptable predictability.

We also explored a comparison of the univariate and multivariate classification on predictive performance. Based on the developed models on day 10, all-variable model (Fig. 6d) yielded the most accurate prediction (91% for non-responders, 85% for responders). Though not as good as all-variable model, model based on IFN-γ secretion descriptors (Fig. 6e) performed much better than model based on phenotypic markers (Fig. 6f). Predictions by each individual variable at day 10 and representative plots were shown in Fig. 6g–n. Only a few variables with high VIP scores (CD4Cmax, CD4tau, CD8+, MDSC) showed slight predictability while most single variables yielded extremely biased prediction. Compared to multivariate classification (Fig. 6d, e), no individual variable can yield comparable predictability.

## Discussion

A limitation to the utility of checkpoint immunotherapy is the lack of predictive biomarkers to guide patient selection. Most studies in the development of diagnostic biomarkers focused on tumour microenvironment.^8,9,31,32^ Many mechanism-based biomarkers like PD-L1 expression and intratumoural lymphoid infiltration have been evaluated in tumour biopsy samples.^31^ Although certain predictability has been obtained, the significant variance in IHC antibody, staining conditions, and anatomical sites have dramatically compromised the reliability and practical applications. Furthermore, both PD-L1 expression and antitumour immune reactivity are dynamic and constantly evolving in respond to immune perturbation.^9^ Our data observed a significant increase of PD-L1 density induced by checkpoint blockades, even in non-responders compared to control (data not shown). Increased PD-L1 expression in serial tumour biopsy was also reported during the atezolizumab therapy.^32^ Therefore, the dynamic nature of the immune system extremely challenges robust predictions by univariate and static biomarkers in the microenvironment. Instead, we addressed this problem by introducing a proof-of-concept biomarker based on the dynamic metrics obtained from the secretion kinetics of IFN-γ by peripheral T cells.

Tumour rejection and extended survival in B16F10 melanoma-bearing mice were observed in a subset of mice undergoing PD-1 blockade (Fig. 1). At the same time, our data suggested large inter-animal variations in tumour growth and intratumoural cell populations (Fig. 1d and S1). Consequently, mice receiving checkpoint blockade were pooled and divided into responder and non-responders according to durations of survival (Fig. 1b).

To allow diagnostic prediction at early stage, predictive variables from circulating lymphocytes in peripheral blood should be more attractive, which provide a non-invasive and real-time strategy. Actually, peripheral lymphocytes have been evidenced to provide valuable insights into patient-specific antitumour response. Alice et al.’s study^33^ found that peripheral PD-1+CD8+ T cells indicate tumour-specific CD8+ T cells and are associated with immune responses induced by therapies blocking the PD-1 pathway in lung cancer patients. Alena et al. also identified neoantigen-specific lymphocytes in the peripheral blood of melanoma patients.^34^ Clinically, increases of absolute lymphocyte counts and percentages of CD4+ and CD8+ T cells were associated with positive outcome of melanoma patients treated with ipilimumab.^35^ Indeed, our data from peripheral phenotypic markers also indicated that lymphocytes in responders at day 10 may experience similar transitions as TILs except CD8+% (Fig. 2 and S1). Although some significant differences were shown, no phenotypic marker can be used to precisely predict individual response due to considerable overlap between responders and non-responders (Fig. 2).

We further explored the predictability of peripheral IFN-γ secretion characteristics. Previous studies highlighted the importance of IFN-γ as a critical regulator in immune response. IFN-γ can directly induce tumour death by means of apoptosis and facilitates CD8+T cell priming, NK cells, and macrophage recruitment and activation.^36^ Adaptive up-regulation of PD-L1 by tumour cells is also induced by enhanced IFN-γ exposure.^3^ On the other hand, the deficiency of IFN-γ signalling impaired tumour rejection in melanoma-bearing mice as well as non-responder patients.^37^ Taken together, we assume that the profile of IFN-γ secretion from lymphocytes may provide a window to evaluate not only their cytotoxic effectiveness but also functional adaptability of immune systems in response to checkpoint immunotherapy.

Conventionally, IFN-γ secretion level has been applied to directly correlate to clinical outcomes.^38^ We alternatively examined multiple descriptors of secretion kinetics to characterise underlying features of IFN-γ secretion from lymphocytes following stimulation (Figs. 3 and 4). Unlike static biomarkers, secretion descriptors comprehensively evaluate secretory capacity (magnitude), speed (time to reach half magnitude, slope), and onset (lag time). A similar strategy was applied to the dynamics of JNK network and successfully predicted survival of neuroblastoma patients.^14^ Dynamics of key factors in apoptotic signalling pathways also showed a high predictive value on fractional cell killing in response to chemotherapies or antibodies.^39,40^ Here we assumed that secretion descriptors of IFN-γ are related to the functionality and adaptability of host immune system. Interestingly, responders showed a tendency to trigger CD4+ cell to release IFN-γ earlier (lower CD4tau), faster (higher CD4h), and greater (higher CD4Cmax) versus non-responders at day 10 (Fig. 4e–h). These features indicate the activated immune status and strong cytotoxic effectiveness. Besides, the strong and prompt IFN-γ release may help to prevent adaptive resistance of tumour cells induced by IFN-γ secreted by T cells.

Even though IFN-γ secretion descriptors conceive valuable diagnostic information, neither individual IFN-γ secretion descriptor nor individual phenotypic marker alone can precisely predict responders (Fig. 6g–n). As critical immune transitions are highly sensitive to initial conditions,^11^ the difference obtained at pre- and early-treatment stage, although small, was still of great predictive value. Therefore, multiple dynamic variables were applied to describe the IFN-γ secretion in a high dimensional manner, multivariate analysis was then used to reduce the dimensionality^25,26^ into a single biomarker by unravelling the latent predictive information. Initially we sought to quantitatively link individual tumour sizes or survival duration with the dynamics of biomarkers using the OPLS regression model. Despite moderate correlation between the variable component (*t*1) and individual responses (*y*1; Fig. S5A and B), samples were not well clustered (Fig. S5D and E), indicating that an accurate prediction of tumour sizes or survival duration was challenging by using the dynamics of biomarkers. It is noteworthy that high and low responses were generally separated by variable components, especially the response denoted as the survival duration (Fig. S5E). Thus, we simplified the OPLS regression model to predict categorical responses (responders and non-responders), which conferred improved model predictability and robustness (Fig. S5C and F).

Indeed, our further studies showed that the categorical OPLS-DA model is capable of extracting combinations of markers and shown to be the most informative method to discriminate responders from non-responders (Fig. 5). Three critical observations were obtained throughout our analysis. Firstly, the regression models based on the dynamic descriptors of IFN-γ secretion performed much better than those on static phenotypic markers (Figs. 5d, e and 6e, f). As shown in Fig. S4E and F, secretion descriptors dominantly accounted for the discrimination, like CD4Cmax, CD8Tc50, and CD4tau. Of note, variable changes from day 3 to day 10, another type of derived “dynamic variables”, also showed high predictability (Fig. 5g). Secondly, the predictive power of the developed model is time dependent. In general, the models built on the data right after treatment (day 10) displayed higher predictive power than that on pre-treatment data (day 3), suggesting that the evolving immune system after checkpoint blockades conveyed high predictive information (Fig. 5b and e, 5c and f). Lastly, multivariate classification far exceeded univariate classification. There was no prediction acceptable based on univariate classification (Fig. 6). Similarly, the models jointly employed IFN-γ secretion descriptors and phenotypic markers achieved better separations (Figs. 5a–f and 6d–f). As expected, the OPLS-DA model after integration of all variables at day 3 and day 10 yielded the most accurate prediction (91% for non-responders, 90% for responders; Fig. 5h). In considering of model complexity and translational challenge, however, model based all variable at day 10 is fully qualified for response discrimination (Figs. 5f and 6d).

Ideal biomarkers for companion diagnosis would be accurate, real-time, and non-invasive. Static biomarkers, even upon serial biopsies, would not meet these criteria. By contrast, our analysis was focused on peripheral blood, which allows real-time monitoring. Moreover, dynamic metrics-based biomarkers are expected to be relatively robust and adaptive to reflect the dynamic nature of evolving immune system. However, there are still some limitations in our current study. Our dynamic biomarker requires simultaneous qualification of multi-variables, which is more labour-consuming than single static biomarker. In the external validation, the compromised predictive performance may largely result from the absence of MDSC% in phenotypic markers. Besides, the diversity of cytokine release was also found as important indicators for immune response.^22^ If the secretory profiles of other T cell-related cytokines, like IL-2 and TNF-α could be characterised simultaneously, our OPLS-DA model is expected to be further optimised.

In conclusion, evaluation of IFN-γ secretion kinetics by peripheral lymphocytes possesses high diagnostic predictabilities of responders to anti-PD1 immunotherapy. Such dynamic metrics-based biomarkers hold promise to improve current companion diagnosis for checkpoint immunotherapies.

## Supplementary information

Supplemental material
