# Correction: Dynamic metrics-based biomarkers to predict responders to anti-PD-1 immunotherapy

**DOI:** 10.1038/s41416-019-0418-5

**Published:** 2019-03-04

**Authors:** Can Liu, Hua He, Xiaobing Li, Maureen A. Su, Yanguang Cao

**Affiliations:** 1 Division of Pharmacotherapy and Experimental Therapeutics, Eshelman School of Pharmacy, University of North Carolina at Chapel Hill, Chapel Hill, NC 27599 USA; 2 School of Pharmacy, China Pharmaceutical University, 210009 Nanjing, Jiangsu China; 3 Department of Pharmacy, Shengjing Hospital of China Medical University, 110004 Shenyang, Liaoning China; 4 Department of Microbiology, Immunology and Medical Genetics (MIMG) and Pediatrics, University of California, Los Angeles, CA 90095 USA; 5 Lineberger Comprehensive Cancer Center, University of North Carolina at Chapel Hill, Chapel Hill, NC 27599 USA

**Keywords:** Cancer immunotherapy, Cancer immunotherapy, Statistical methods, Cancer immunotherapy, Statistical methods

**Correction to:** *British Journal of Cancer* (2019) **120**, 346–355; 10.1038/s41416-018-0363-8; www.bjcancer.com; published online 27 December 2018.

Since the publication of this paper, the authors noticed that the funding information for Maureen A. Su was not included. Therefore the authors would like to add the following information to the Acknowledgements section:

