# Supplementary material for: Dynamic metrics-based biomarkers to predict responders to anti-PD-1 immunotherapy
Source: Br J Cancer. 2018 Dec 27;120(3):346–55. doi: 10.1038/s41416-018-0363-8 (PMC6353899; doi:10.1038/s41416-018-0363-8)

**Supplementary Figures for MD-2018-3215R**

Figure S1 Cell phenotyping on tumor-infiltrating lymphocytes (TILs) from an independent experiment on day 17 following tumor inoculation. Percent of CD45.2+ TILs composed of (A) CD4+ T effector cells, (B) CD8+ T effector cells and (E) CD25+Foxp3+ Treg are shown. The proportion of PD1+ expressing cells in CD4+ and CD8+ gated TILs were shown in (C) and (D) respectively. The ratios of (F) CD8+ T cells to Treg are calculated. Values shown are for individually analyzed mice. Data distribution was described by boxplot (minimum, first quartile, median, third quartile, and maximum). Two-group differences are determined between cIg vs. treatment (responders plus non-responders) and responders vs. non-responders by Wilcoxon-Mann-Whitney test (* p<0.05).


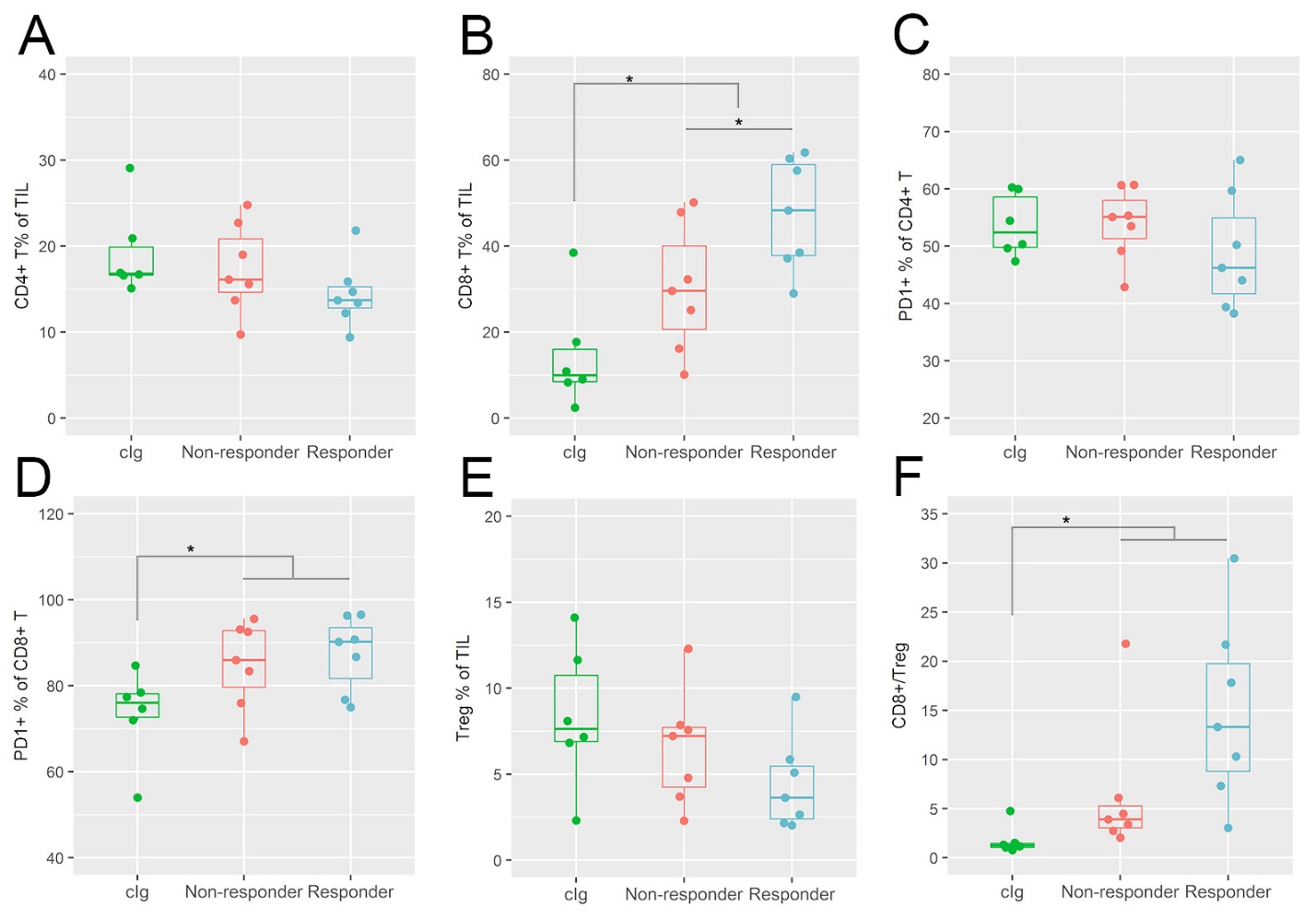


Figure S2 Flow cytometric analysis of CD4+, CD8+, CD25+Foxp3+ Treg, CD8+PD-1+ and Gr-1+CD11b+ MDSC cells in peripheral blood from representative responder and non-responder.


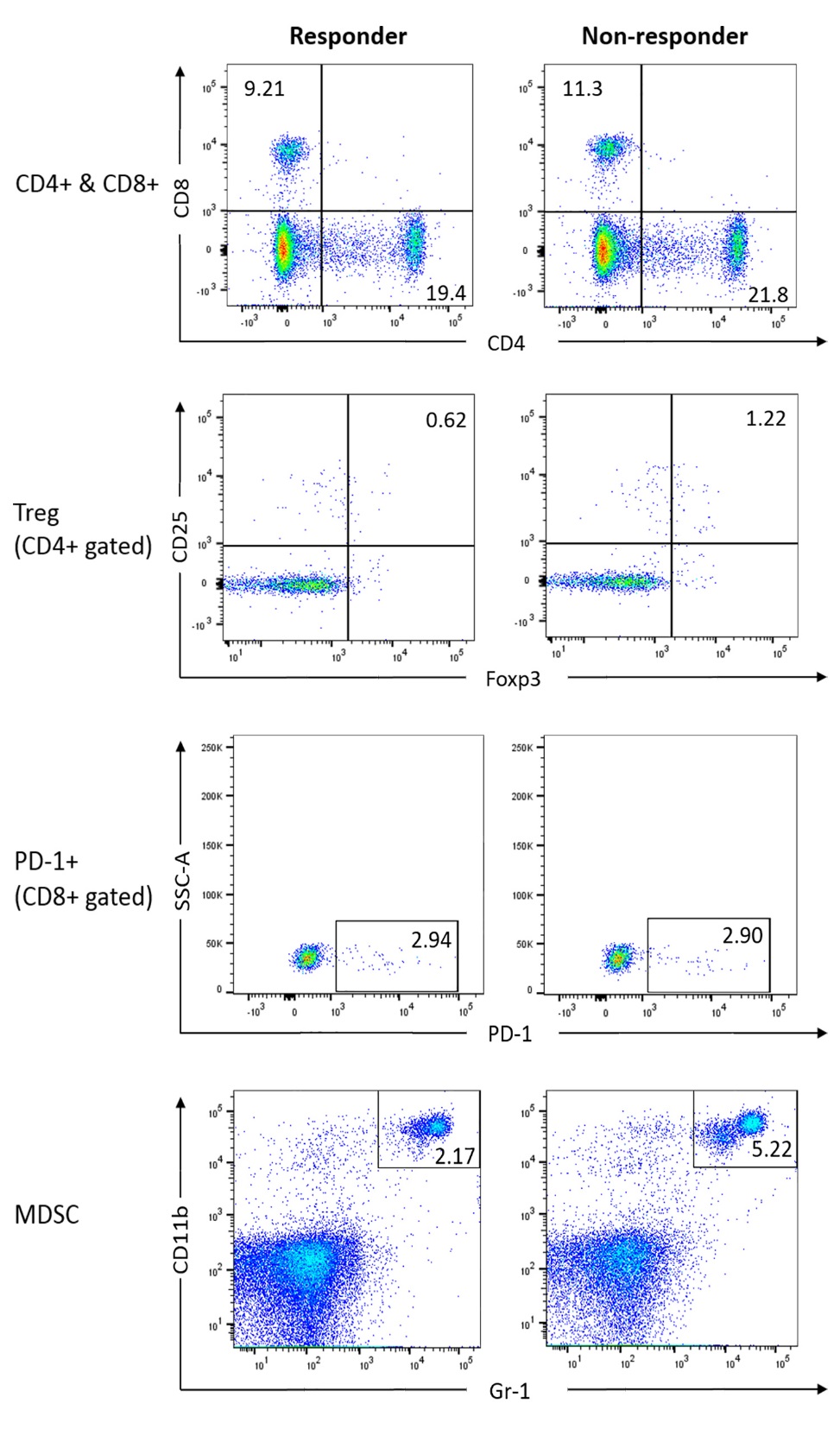


Figure S3 Goodness-of-fit plots of predicted versus observed IFN-γ secretion from CD4+ (A, B) and CD8+ (C, D) cells at day 3 (A, C) and day 10 (B, D). The solid black and grey line indicate identity line and linear regression fit respectively.


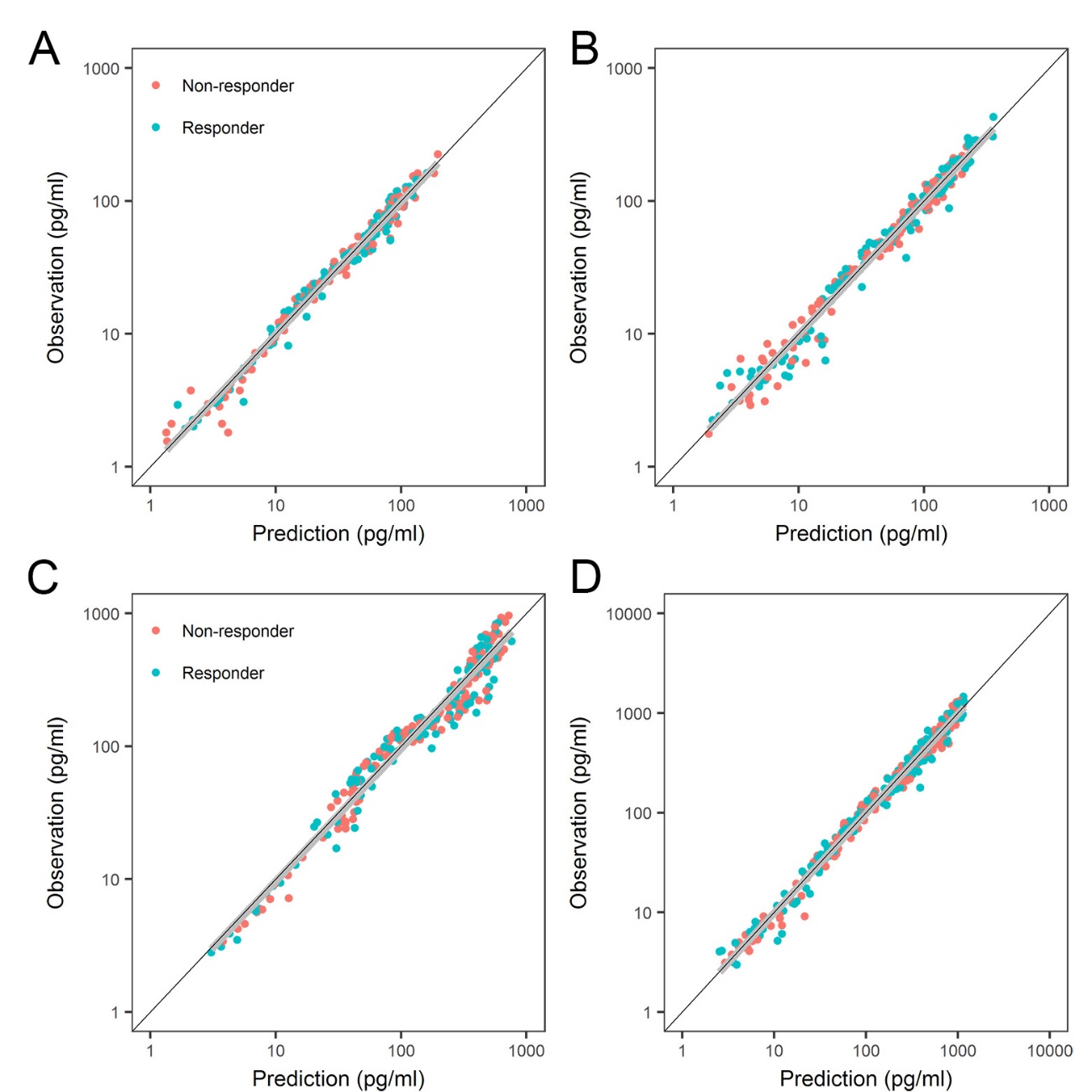


# Figure S4 Validation plots (A, D), VIP plots (B, E) and loading plots (C, F) of models shown in Fig 5G (A-C) and 5H (D-F). Two-hundred random permutations were performed in validation plots, and the resulting R^2^Y and Q^2^Y values were shown as dots. The horizontal axis shows the correlation between the permuted and actual y matrix. ∆ means the variable changes from day 3 to day 10. CD4+, CD8+, Treg, MDSC represent phenotypic markers (cell density of CD4+, CD8+, Treg and MDSC cells in peripheral blood respectively). PD1+CD4+ and PD1+CD8+ stand for frequency of PD1+ subset in CD4+ and CD8+ cells respectively. CD4Cmax, CD4Tc50, CD4h and CD4tau indicate IFN-γ secretion descriptors (C_max_, Tc50, h and tau) derived from CD4+ lymphocytes. CD8Cmax, CD8Tc50, CD8h and CD8tau indicate IFN-γ secretion descriptors (C_max_, Tc50, h and tau) derived from CD8+ lymphocytes.


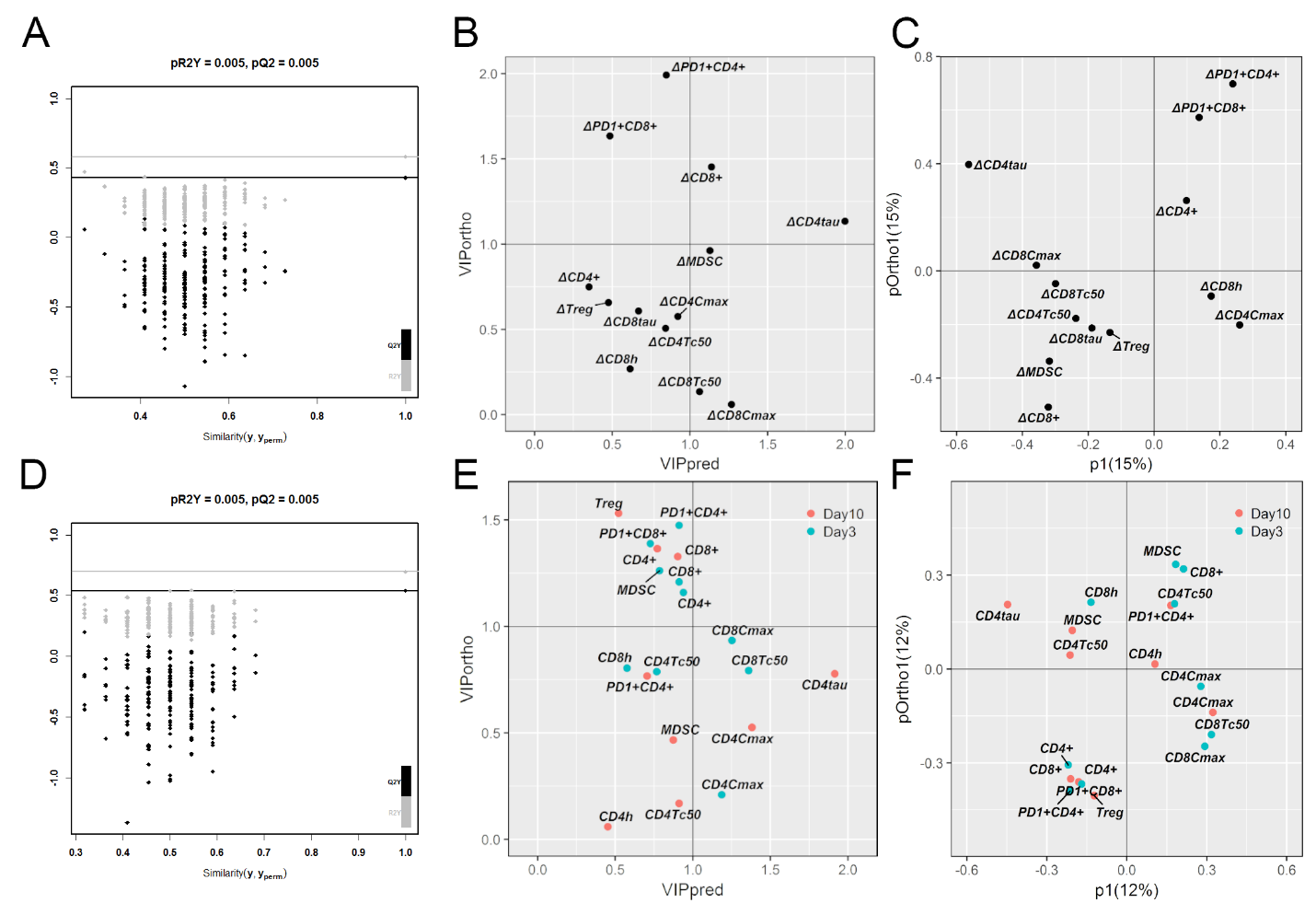


Figure S5 OPLS model outcomes for distinguishing individual responses to anti-PD-1 checkpoint inhibitor. A-C: Variable correlations with the components. D-F: score plots for optimized OPLS models. Tumor size at day 14 (A, D), survival duration (B, E), and categorical response (C, F) were used as response matrix (y1) respectively. Secretion descriptors and phenotypic markers at day 10 were used as predictor matrix (X vars). The model was generated using one predictive component (t1) and the first orthogonal component (to1). Each dot in score plots stands for OPLS score of individual mouse. R^2^X and R^2^Y: percentage of predictor and response variance explained by the model. Q^2^Y: model predictive performance estimated by cross validation. RMSEE: root mean square error of estimation.


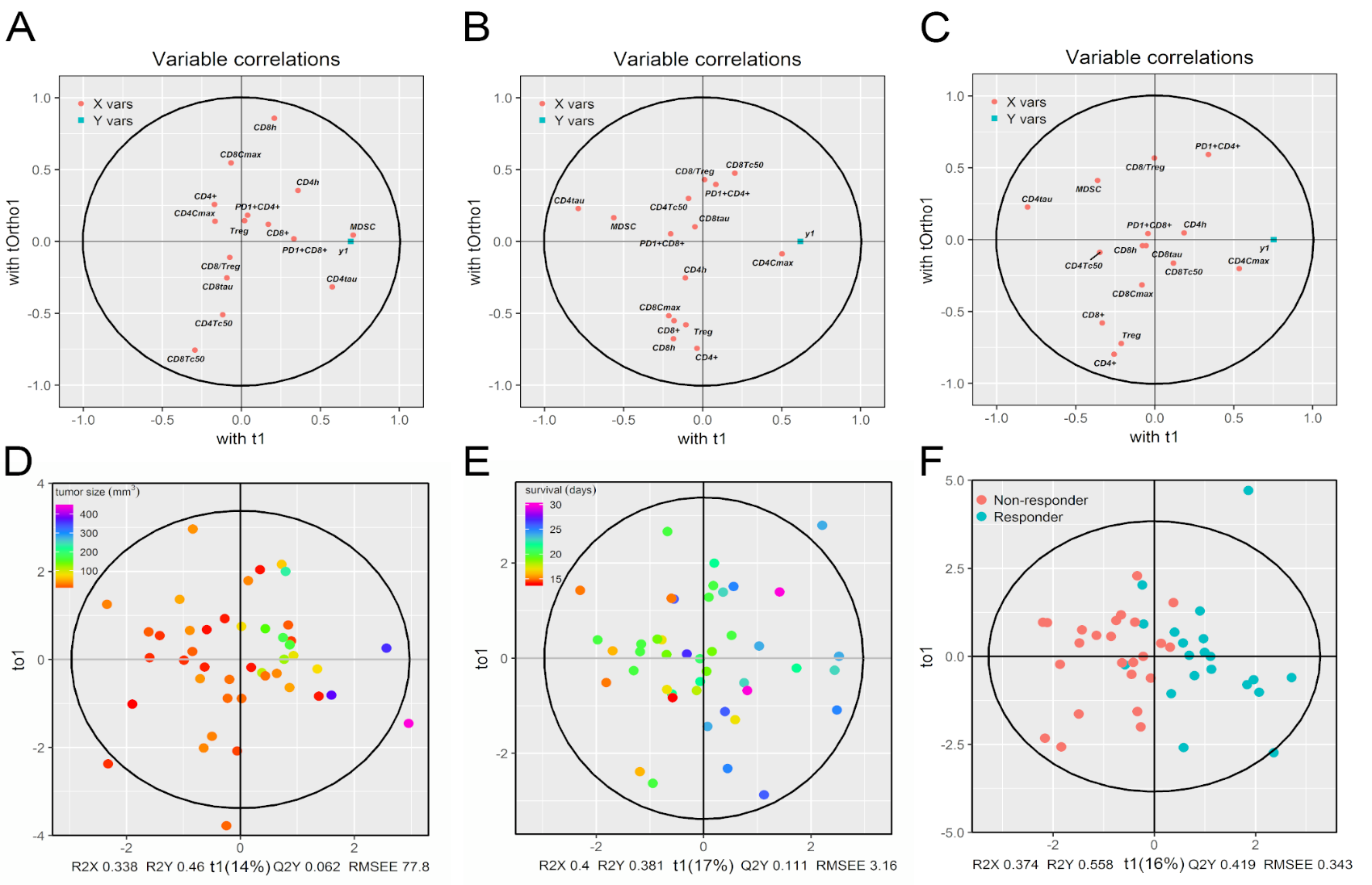

Supplement: Supplementary file 1 — Supplemental material [file 41416_2018_363_MOESM1_ESM.docx]
